# Supplementary material for: Improving human activity classification based on micro-doppler signatures of FMCW radar with the effect of noise
Source: PLoS One. 2024 Aug 1;19(8):e0308045. doi: 10.1371/journal.pone.0308045 (PMC11293691; doi:10.1371/journal.pone.0308045)
Supplement: Attachment — Submitted filename: Response to Reviewers.doc [file pone.0308045.s001.doc]

**Original Manuscript ID:** PONE-D-24-04469

**Original Article Title:** “Improving Human Activity Classification Based on Micro-Doppler Signatures of FMCW Radar with the Effect of Noise”

**To:** Reviewer

**Re:** Response to reviewer

Dear Reviewer,

We appreciate you for your precious time in reviewing our paper and providing valuable comments. It was your valuable and insightful comments that led to possible improvements in the current version. The authors have carefully considered the comments and tried our best to address every one of them.

We are uploading our point-by-point response to the comments, an updated manuscript with blue highlighting indicating changes, and a manuscript without track changes.

Note: In the "Response to Reviewers", the black text is the author's explanation and actions, and the blue text is the content that was added and revised in the "Revised Manuscript with Track Changes".

Best regards,

Reviewer #1:

**1. The authors proposed a method to classify human movements using radar spectrograms. In the case of a radar spectrogram, its characteristics change depending on the distance between the person and the radar sensor, the angle between the person and the radar sensor, etc. It is essential to conduct additional experiments considering various conditions.**

**Author response:** The authors would like to thank the Reviewer for your constructive comment.

Indeed, m-D characteristics shown on radar spectrogram will change depending on the distance and aspect angles between the person and the radar sensor. Figure 1 and Figure 2 display the m-D signatures of two activities, walking and walking to fall at various distances and aspect angles. When adding different SNR levels, the m-D signatures are almost completely submerged under the background noise and are difficult to observe. Therefore, the authors used the initially measured spectrograms (without adding different SNR levels) to easily observe the m-D signatures displayed on the spectrogram. Observing in detail, the m-D signatures on the spectrogram are presented completely differently when the aspect angle is set to [0, 45^0^, -45^0^, 90^0^, -90^0^]. However, when the distance is changed, these signatures are almost the same; the spectrogram only shows changes in the energy levels. An observed target that is closer to the radar will have a higher energy level, and vice versa.

The radar equation below explains why the energy level and the m-D signatures on the spectrogram change as the distance and aspect angle between the target and the radar change.

$$P_{r}=\frac{P_{t}G_{t}\delta}{\left(4\pi R^{2}\right)^{2}}\frac{G_{r}\lambda^{2}}{4\pi}=\frac{P_{t}G_{t}G_{r}\lambda^{2}\delta}{(4\pi)^{3}R^{4}}[W]$$

where, $P_{r}$ is the received power at the radar receiver, $P_{t}$ is the transmitted power, $G_{t}$ and $G_{r}$ are transmitter and receiver antenna gains, respectively, $\lambda$ is the wavelength, $\delta$ is radar cross-section of the target, and $R$ is the distance between the radar and the target.

It is clear that *R* is inversely proportional to the power received by the receiver. Therefore, as the distance between the targets increases, the received power decreases, resulting in a lower energy level on the spectrogram. Moreover, the aspect angle represents the direction of the target when acting relative to the radar position. These aspect angles will have an impact on the target's effective radar cross-section. Different aspect angles will lead to different radar cross-sections. Based on the above radar equation, this will change the received power at the radar receiver, thereby changing the m-D signatures on the obtained spectrogram.

Figure 1. Spectrograms of walking action at various distances and aspect angles.

Figure 2. Spectrograms of walk to fall action at various distances and aspect angles.

**Author action:** Based on the reviewer's comments and the above discussion. The author added experimental results when changing distance and aspect angles in Section " Performance of the proposed model when changing the distance and aspect angles between object and radar”. The revised content is highlighted in blue on pages 19 and 20 of the “Revised Manuscript with Track Changes”, as follows:

“To evaluate the proposed model's performance when changing the distance and aspect angles between the object and radar, we have built a supplementary dataset consisting of varying distances: 2m, 3m, 5m, and 7m. The aspect angles remain consistent at [0^0^, 45^0^, -45^0^, 90^0^, -90^0^]. The additional dataset will consist of 13200 samples, which will include 4 distances, 11 actions, 10 iterations, 5 aspect angles, and 6 SNR levels. The additional dataset is randomly divided into 80% for training, and the remaining 20% is used for testing. The transfer learning method is applied to the CRCNN model that was trained with the initial dataset.

1. Comparison results with various distances

To evaluate the classification accuracy in detail when the distance between the target and the radar sensor changes, the trained CRCNN model is trained with all distances (2m, 3m, 5m, and 7m) and then tested with each different distance. Table 1 displays the classification accuracy at different distances. The classification accuracy varies with the changing distance between the radar and the target; however, this variation is insignificant (it just fluctuates in the range from 2% to 3%). This result demonstrates the model's capability to accurately categorize targets even when there is a variation in the distance between the target and the radar.

Table 1. Classification accuracy with various distances

| **Distances** | Accuracy (%) | | | | | | |
| --- | --- | --- | --- | --- | --- | --- | --- |
|  | -15dB | -10dB | -5dB | 0dB | 5dB | 10dB | **avg** |
| 2m | 83.47 | 92.56 | 93.39 | 97.52 | 98.35 | 99.17 | **94.08** |
| 3m | 81.82 | 91.74 | 94.21 | 96.69 | 97.52 | 98.35 | **93.39** |
| 5m | 78.32 | 87.41 | 91.61 | 93.71 | 96.5 | 97.2 | **90.79** |
| 7m | 77.62 | 86.71 | 90.91 | 92.31 | 95.8 | 96.5 | **89.98** |

2. Comparison results with various aspect angles

Figure 3. Classification accuracy with various aspect angles.

To evaluate the classification accuracy in detail when the aspect angle between the target and the radar sensor changes, the trained CRCNN model is trained with three separate datasets containing different aspect angles and then tested with different aspect angles in turn. In particular, the first dataset only contains data with aspect angle of $0^{\circ}$ between the person and radar sensor; the second dataset contains aspect angles of $0^{\circ}$ and $\pm45^{\circ}$; and the third dataset contains aspect angles of $0^{\circ}$, $\pm45^{\circ}$, and $\pm90^{\circ}$. Figure 3 illustrates the classification accuracy across various aspect angles using three distinct training datasets. The first training dataset, comprising data solely from a $0^{\circ}$ aspect angle (depicted by the blue line), achieves the highest accuracy at approximately 94% at a $0^{\circ}$ angle. Conversely, accuracy greatly decreases for other angles due to a lack of training, ranging from 76% to 81%. The second training dataset, which encompasses data from $0^{\circ}$ and $\pm45^{\circ}$ angles (illustrated by the black line), notably enhances accuracy, particularly for the $45^{\circ}$ and $-45^{\circ}$ angles, with an improvement of up to 10%. However, the observed accuracy at angles $90^{\circ}$ and $-90^{\circ}$ is still relatively low. Finally, the inclusion of all five aspect angles in the third training dataset (represented by the red line) improves accuracy at $90^{\circ}$ and $-90^{\circ}$ angles. These findings underscore the impact of aspect angles on classification accuracy and highlight the importance of training datasets.”

**2. I am curious whether the number of layers, number of filters, and size of filters in the deep learning structure in Figure 6 used by the authors have been optimized. There is a need to justify why the authors set the hyperparameters in the structure as shown.**

**Author response:** The authors would like to thank the Reviewer for your constructive comment.

The authors experiment by changing different hyperparameters of the model, such as, the number of layers, the number of filters, and the size of the filters, to find the best model that balances classification accuracy, complexity, and prediction time. As a result, the proposed CRCNN structure is illustrated in Figure 7 (page 10, in manuscript).

Specifically, the results in Figure 8 and Table 2 (in pages 12 and 13, section "The effect of the number of filters on the performance of the proposed model", in manuscript) explain why the number of filters per convolution layer is chosen to be 64. The results in Figure 9 (in page 14, section "The effect of the number of cross-residual connections on the performance of the proposed model", in manuscript) explain why the number of C-R connections is chosen to be 6.

In addition, to explain the reason for choosing two filters with different sizes, 3x3//5x5, in the im-res block, we have added some results. Table 2 shows additional results when the sizes of the two parallel convolution layers are modified to 3x3//3x3, 3x3//7x7, and 5x5//7x7, respectively.

**Author action:** Based on the reviewer's comments and the above discussion. The author added experimental results when changing the filter size in the im-res block in Section "The effect of the filter size in the im-res block on the performance of the proposed model”. The revised content is highlighted in blue on page 13 of the “Revised Manuscript with Track Changes”, as follows:

“Table 2 displays the comparison results for accuracy when changing the size of filters in im-res blocks. The model, which uses 2 parallel convolutional layers with a fixed size of 3x3//3x3, has the fastest prediction time, but the classification accuracy is the lowest (just 85.61%). Models using filters of sizes 3x3//7x7 and 5x5//7x7 achieve accuracies of 88.36% and 87.96%, respectively, but they come at the cost of a sharp increase in learned parameters and prediction time. With a selected size of 3x3//5x5, the CRCNN model achieved the highest accuracy (at 89.11%) and a prediction time of 13.1 ms. This result has proven that using filters with different sizes will help extract both local and global features more optimally, and 3x3 and 5x5 are the appropriate sizes for the features to be extracted from the input dataset in the proposed CRCNN model.”

Table 2. Compare results with different size filters in the im-res block

| Filter size | Accuracy (%) | | | | | | | Para  (M) | Train  (m) | Pre  (ms) |
| --- | --- | --- | --- | --- | --- | --- | --- | --- | --- | --- |
|  | -15dB | -10dB | -5dB | 0dB | 5dB | 10dB | avg |  |  |  |
| 3x3//3x3 | 69.39 | 83.18 | 84.55 | 87.27 | 93.64 | 95.61 | **85.61** | 0.8 | 59 | 12.9 |
| 3x3//5x5 | 74.85 | 86.21 | 87.27 | 91.61 | 96.36 | 98.33 | **89.11** | 1.2 | 61 | 13.1 |
| 3x3//7x7 | 75.76 | 85.3 | 85.61 | 90.61 | 95.3 | 97.58 | **88.36** | 1.9 | 67 | 13.7 |
| 5x5//7x7 | 73.03 | 85.91 | 86.06 | 89.55 | 95.3 | 97.88 | **87.96** | 2.4 | 71 | 14.4 |

**Note:** Para is the learnable parameters, Train is training time and Pre is the prediction time; (M denotes million; m and ms denote minute and millisecond, respectively).

**3. The authors compared classification performance with other deep learning techniques. In addition to classification accuracy, it would be a good idea to compare calculation complexity, calculation time, and time required for data training.**

**Author response:** The authors would like to thank the Reviewer for your constructive comment.

In this paper, the authors compare the average classification accuracy, prediction time, and time required for data training. According to the reviewer's contributions, we will add additional layers, learnable parameters, and three additional important metrics, precision, recall, and F1-score, to evaluate the denoising effectiveness of the classifiers.

**Author action:** Experimental results are added in Section " Comparison results on the noise-added dataset”. The revised content is highlighted in blue on pages 14 and 15 of the “Revised Manuscript with Track Changes”, as follows:

“Table 3 shows the results of the proposed model compared to other DCNN models. DIAT is a lightweight self-custom network (the learnable parameter is just 2 M), so the extracted features can be confused with the extracted features from the added-noise dataset. This led to the accuracy of DIAT being the lowest (just nearly 70%). Although RepVGG has the most learnable parameters (24.6 M), it provides low classification accuracy (around 76%) and has the longest processing time (approximately 15.4 ms). This is because RepVGG is designed with a fixed filter size of 3 × 3, which cannot provide a multi-scale of spatial regions in the m-D spectrogram. In contrast, the number of parameters to be learned by the CRCNN model is the lowest, at around 1.2 M, while the correct classification rate produced by this model is the greatest, at approximately 89.1%. This result can be attributed to the obtained feature diversity and the effective reusability of former features from the previous layers. Furthermore, the prediction time of the suggested network is 13.1 ms, which is comparable to ResNet and DINN and significantly faster than MobileNet-V2 (14.25 ms) and Dop-DenseNet (14.84 ms).

Table 3. Comparison results of CRCNN with state-of-the-art CNN models

| Classifiers | Acc  (%) | Para  (M) | Layers | Train  (m) | Pre  (ms) |
| --- | --- | --- | --- | --- | --- |
| DIAT | 69.9 | 2 | 76 | 146 | 14.6 |
| DINN | 72.5 | 1.7 | 129 | 93 | 13.3 |
| RepVGG | 75.4 | 24.6 | 117 | 86 | 15.4 |
| DopDense | 82.1 | 1.7 | 49 | 76 | 14.84 |
| ResNet | 87 | 11.1 | 71 | 72 | 13.4 |
| MobileNet-V2 | 87.9 | 2.2 | 154 | 100 | 14.25 |
| Proposed | 89.1 | 1.2 | 87 | 61 | 13.1 |

Reviewer #2:

**1. Does the abstract clearly summarize the main contributions and findings of the study?**

**Author response:** The authors would like to thank the Reviewer for your constructive comment.

**Author action:** The abstract is edited and clearly summarizes the main contributions of the study in Section “Abstract”. The revised content is highlighted in blue on page 1 of the “Revised Manuscript with Track Changes”, as follows:

“Nowadays, classifying human activities is applied in many essential fields, such as healthcare, security monitoring, and search and rescue missions. Radar sensor-based human activity classification is regarded as a superior approach in comparison to other techniques, such as visual perception-based methodologies and wearable gadgets. However, noise usually exists throughout the process of extracting raw radar signals, decreasing the quality and reliability of the extracted features. This paper presents a novel method for removing white Gaussian noise from raw radar signals using a denoising algorithm before classifying human activities using a deep convolutional neural network (DCNN). Specifically, the denoising algorithm is used as a preprocessing step to remove white Gaussian noise from the input raw radar signal. After that, a lightweight Cross-Residual Convolutional Neural Network (CRCNN) with adaptable cross-residual connections is suggested for classification. The analysis results show that the denoising algorithm with a range-bin interval of 3 and a cut-threshold value of 3 achieves the best denoising effect. When the denoising algorithm was applied to the dataset, CRCNN improved the right classification rate by up to 10% compared to the recognition results achieved with the original noise-added dataset. Additionally, a comparison of the CRCNN with the denoising algorithm solution with six cutting-edge DCNNs was conducted. The experimental results reveal that the proposed model greatly outperforms the others.”

**2. Is the significance of the proposed approach in the context of existing challenges in human activity classification using radar sensors adequately highlighted?**

**Author response:** The authors would like to thank the Reviewer for your constructive comment.

Some challenges remain for radar-based human activity classification, including:

- The classification accuracy in a real environment with noise effect.

- Data collection challenges: collecting data from radar sensors can be limited by factors such as distance and aspect angles.

- Diverse types of activities: Humans engage in a wide range of activities, from walking to sitting, standing, etc. Accurately recognizing these activities can be challenging.

The proposed study has emphasized addressing the above-mentioned challenges.

Firstly, the main focus of this study will be on denoising in both directions. This includes a suggested algorithm for removing white Gaussian noise from noisy raw data during the preprocessing step. Next, a self-designed network will be used to reduce computational complexity and improve classification accuracy.

Secondly, build the dataset with various aspect angles [0^0^, 45^0^, -45^0^, 90^0^, -90^0^] to better suit the reality of radar data collection. (this content is presented on pages 5 and 6, section "Data preparation", in manuscript)

Thirdly, collecting data on 11 daily human activities: walking, body rotation, punching, kicking, grabbing, standing up, sitting down, standing to walk, walking to sit, walking to fall, and falling to walk to enrich the daily activities instead of just using some simple activities such as walking, sitting down, standing up, etc. (this content is presented on pages 5 and 6, section "Data preparation", in manuscript)

**Author action:** Further emphasis and clarification on the proposed denoising algorithm is mentioned in Section “Introduction”. The revised content is highlighted in blue on page 3 of the “Revised Manuscript with Track Changes”, as follows:

“Both of the above research directions have effectively improved classification accuracy. However, significant challenges remain for radar data processing, including the effective classification of varied human activities in real environments with noise effects. Noise exists throughout the process of receiving radar signals, decreasing the quality and reliability of extracted features from radar signals [15, 16]. Under the influence of noise, the extracted features to classify these actions are greatly submerged in the background, which leads to a significant decline in classification accuracy. Therefore, denoising techniques play an important role in improving the classification accuracy of human activities from radar signals.”

**3. How does the proposed approach differ from existing methods in handling environmental noise in radar-based human activity classification?**

**Author response:** The authors would like to thank the Reviewer for your constructive comment.

To respond to the reviewer's comments, the author has added and clarified some contents and results, as follows:

Firstly, introduce and analyze some existing denoising methods.

Second, the main contributions of the proposed denoising method are detailed and highlighted.

Thirdly, add experimental results comparing the proposed denoising algorithm with existing denoising algorithms.

**Author action 1:** The first and second content are edited in Section "Introduction". The revised content is highlighted in blue on pages 3 and 4 of the “Revised Manuscript with Track Changes”, as follows:

“Various denoising methods have been developed for the radar signal, such as wavelet denoising, threshold filter, adaptive threshold filter, and high‐resolution spectral analysis [17–19]. Besides, denoising in machine vision has made rapid progress [20], there is also extensive research focused on signal denoising obtained from vision through deep neural networks. Model-based denoising methods address the issue of additional white Gaussian noise using designs like U-Net [21] and residual learning [22]. Model-based denoising methods have had significant noise reduction results, however, the input noisy images are mainly digital images, the denoising of input signals as complex signals (In-Phase and Quadrature—I & Q) is still limited. Moreover, the importance of signal pre-processing has been almost forgotten due to too much reliance on machine learning to find patterns for recognition. Preprocessing methods, especially denoising techniques, need to be introduced before the classification model with the aim of improving the reliability and accuracy of the classification. Therefore, the major research in this study will focus on both directions, including a proposed algorithm to eliminate white Gauss noise from the noisy raw data at the preprocessing step and a novel lightweight self-designed network to reduce computational complexity and improve classification accuracy. The key contributions of this paper can be summarized as follows:

Firstly, propose a denoising algorithm accomplished through two primary steps: determine the optimal range-bin interval using minimum entropy information [23-25], then identify an appropriate cut-threshold value through experimentation to remove white Gauss noise from the input noising signal.

Secondly, a novel customized lightweight CNN, namely Cross-Residual Convolutional Network (CRCNN), is proposed for classifying indoor human activities based on m-D signatures obtained from the denoised spectrograms. The two main innovations of the proposed model show as follows:

- These filters of CRCNN have different sizes, including 1x1, 3x3, and 5x5, which are intelligently designed to be able to extract the most diverse features from previous feature maps, including global and local distribution.

- Cross-residual connections are employed appropriately in im-res blocks to reuse previously useful feature maps that may be lost when going through the model's extraction core flow.

The classification accuracy of CRCNN outperforms six existing state‐of‐the‐art DCNNs and is validated by comparing it with RepVGG [26], Dop-DenseNet [27], MobileNet-V2 [28], ResNet [29], DINN [14], and DIAT-RadHARNet (DIAT) [30] with varying signal‐to‐noise ratios to demonstrate its robustness.

Finally, the proposed approach is evaluated and compared with several existing denoising methods. The results demonstrate that the average classification accuracy improves by up to 10% compared to the original noisy dataset and reaches above 99% at a -10 dB noise level.”

**Author action 2:** The experimental results comparing the proposed denoising algorithm with existing denoising algorithms are added in Section "Comparison results of the proposed denoising method with others". The revised content is highlighted in blue on pages 16 and 17 of the “Revised Manuscript with Track Changes”, as follows:

Figure 4. The spectrograms of different denoising methods for walking action.

Figure 4 shows the denoising spectrogram of denoising methods. Figure 4a shows spectrogram at different SNR levels; the m-D signatures are almost completely submerged under the noise background at SNR levels less than 0 dB. Although the adaptive threshold (ATh) method has the ability to eliminate noise, the results are not clear and completely fail at SNR levels less than 0 dB. When background noise is completely removed, the PCC-DT method has a better denoising ability than the ATh method at SNR levels greater than 0 dB, but it comes with the trade-off of significantly losing significant valuable m-D signatures. This leads to exceptionally high accuracy at 0 dB SNR but gradually decreases at 10 dB SNR. Furthermore, the choice of threshold value and window length of the Hampel filter has a significant impact on the quality of PCC-DT. These selected values are not suitable for all different SNR levels, so for SNR levels less than 0 dB, this PCC-DT method is not really effective. In addition, the results comparing the classification accuracy of the denoised dataset by the proposed method with other denoising methods by the CRCNN model are shown in Table 4.

Table 4. Comparison results of proposed approach with existing denoising methods.

| **Methods** | **Accuracy (%)** | | | | | | |
| --- | --- | --- | --- | --- | --- | --- | --- |
|  | -15dB | -10dB | -5dB | 0dB | 5dB | 10dB | **avg** |
| Noise | 74.85 | 86.21 | 87.27 | 91.61 | 96.36 | 98.33 | **89.11** |
| ATh [19] | 70.45 | 83.94 | 85.45 | 93.93 | 97.58 | 98.64 | **88.33** |
| PCC-DT [8] | 75.77 | 79.09 | 84.39 | 96.21 | 97.73 | 98.27 | **88.61** |
| Transfer learning [35] | 73.48 | 85.42 | 86.36 | 90.52 | 94.09 | 97.42 | **87.88** |
| Denoising | 98.33 | 99.09 | 99.39 | 99.55 | 99.7 | 99.85 | **99.32** |

Table 4 shows the results of the classification accuracy of the proposed method compared with three other denoising methods, including the ATh, the PCC-DT, and the transfer learning method. The ATh method uses a threshold filter that adapts to different SNR levels to eliminate noise. However, for signals with an SNR level of less than 0 dB, this method is not really effective. A similar result is observed for the PCC-DT method, which uses pattern contour-confined Doppler-time maps to minimize redundant information and remove noise points with high power density. The accuracy was significantly improved at the 0dB-SNR level with an accuracy increase of up to 5% but gradually decreased at the 10dB-SNR level because m-D signatures were filtered during the noise denoising process. In contrast to the two methods mentioned above, the transfer learning method uses the pre-trained model to extract features without using a pre-processing step to remove noise; this result shows the accuracy achieved at different SNR levels is the lowest. The proposed method combines denoising in the preprocessing step with the CRCNN model to achieve the best classification results, outperforming the other methods.

**4. What are the specific limitations of visual perception-based methodologies that the proposed radar sensor solution aims to overcome?**

**Author response:** The authors would like to thank the Reviewer for your constructive comment.

Nowadays, vision-based techniques applied to the current activity classification have many limitations, such as requirements for lighting, constraints in operating distance, dependence on favorable weather conditions, and violation of personal privacy. Using radar can completely overcome all existing disadvantages of vision-based techniques due to its benefits in terms of personal privacy compliance, non-contact sensing, materials penetration, long operating distance, and being unaffected by weather conditions and lighting.

However, to further clarify the advantages of radar compared to existing health monitoring methods, the authors added wearable devices. Then summarize the advantages and disadvantages of the methods in Table 5 to make it easier to observe and compare.

**Author action:** The author adds additional content in Section “Introduction”. The revised content is highlighted in blue on pages 1, 2 of the “Revised Manuscript with Track Changes”, as follows:

“Several health-monitoring gadgets are available to address this issue, such as vision-based techniques [2], wearable devices [3], and radar sensors [4]. In good lighting conditions, vision-based techniques can work well with high accuracy and without affecting user comfort. However, it is greatly affected by surrounding lighting and weather conditions and violates the user's personal privacy [5]. Although wearable devices can completely overcome the disadvantages of vision-based techniques, their most significant drawback is that they must be worn continuously, causing certain discomfort to users, especially when resting or going to bed. Out of all the technologies mentioned, radar stands out due to its superiority in various aspects, as seen in Table 5. Therefore, the utilization of radar systems for human activity recognition is a reasonable choice due to its benefits in terms of personal privacy compliance, non-contact sensing, and being unaffected by weather conditions [6]”

Table 5. Comparison between various health‐monitoring methods

| Gadgets | Lighting  independent | Weather independent | Privacy protection | Comfortable |
| --- | --- | --- | --- | --- |
| Vision-based methods | No | No | No | Yes |
| Wearable devices | Yes | Yes | Yes | No |
| Radar | Yes | Yes | Yes | Yes |

**5. How is the optimal range-bin interval determined using minimum entropy information, and what is the theoretical basis for this approach?**

**Author response:** The authors would like to thank the Reviewer for your constructive comment.

To clarify the theoretical basis for using the minimum entropy criterion to determine the optimal range-bin interval, the authors would like to present the basic concept of entropy, some of its properties, and several research papers that have used this method.

Author action: The author adds additional content in Section “Minimum entropy criterion”. The revised content is highlighted in blue on page 7 of the “Revised Manuscript with Track Changes”, as follows:

“Information entropy, which measures data uncertainty, is a cornerstone of information theory. Higher uncertainty corresponds to higher entropy, while lower uncertainty results in lower entropy levels. In the context of time-frequency distributions, entropy assesses the uncertainty inherent in the frequency distribution at a specific time. For a discrete probability distribution $p=\left(p_{1},p_{2},\cdots,p_{n}\right)$, its entropy is calculated as $H(p)=-\sum_{i} p_{i}\ln p_{i}$, where $p_{i}$ represents the probability of each value. Similarly, in the time-frequency domain, if $p=\left(p_{1},p_{2},\cdots,p_{F}\right)$represents the frequency distribution at a certain time *t*, with the entropy function $H_{t}\left(p_{t}\right)=-\sum_{F} p_{t}\ln p_{t}$ quantifies the uncertainty in the frequency distribution. The entropy function exhibits symmetry and extremum properties. The first property shows that the information entropy remains unchanged when the order of variables in the probability distribution sequence is altered. The second property shows that the information entropy reaches maximum entropy when the energy distribution of frequency resolution units is uniform and minimum when only one frequency unit dominates. This is also an important basis for the algorithm to track the energy peak by using the minimum information entropy.”

**6. Can you provide more details on the architecture of the Cross-Residual Convolutional Neural Network (CRCNN) and the rationale behind its design choices?**

**Author response:** The authors would like to thank the Reviewer for your constructive comment.

**Author action 1:** The author adds a detailed architecture of the Cross-Residual Convolutional Neural Network (CRCNN) in Section “Proposed CRCNN-based classification approach”. The revised content is highlighted in blue on page 10 of the “Revised Manuscript with Track Changes”, as follows:

“The overall architecture of the CRCNN includes three main parts: an input block, the main feature extraction block “Cross-Residual” and an output block. The input block has a size equal to the size of the input spectrogram. The main feature extraction block, called "Cross-Residual," is made up of six C-R connections and seven im-res blocks (shown in Fig. 7, in manuscript). The black line shows the straight propagation, and the red line shows the cross-connection. The output block, which contains a Conv-unit-2, a fully connected (FCN) layer, a softmax layer, and a classification (class) layer, is used to classify the action classes.”

**Author action 2:** The author adds 2 major reasons for choosing CRCNN's design in Section “Proposed CRCNN-based classification approach”. The revised content is highlighted in blue on page 11 of the “Revised Manuscript with Track Changes”, as follows:

“Firstly, the convolutional layers of conventional CNNs or DCNNs usually have a size of 3x3. However, the region containing valuable m-D signatures of various human activities on the input spectrogram has completely different distributions based on the type of activity, distance, and aspect angle of the radar. Therefore, using a filter with a fixed size of 3x3 will not be effective in extracting features, and determining the ideal filter size for extracting and learning those signatures is a challenge. A larger filter yields a global distribution, whereas a smaller one yields a distribution that is limited to a specific locality. Besides, raising the depth is susceptible to overfitting. The answer to this difficulty is to run different-sized kernels on the same input tensor [16]. Therefore, in the structure of the proposed model, filters of different sizes, including 1x1, 3×3 and 5×5, are intelligently designed to be able to extract the most diverse features, including global and local distribution from the same input feature map. Besides, the design can ensure the best balance between classification accuracy and computational complexity when using more than two conv-unit as well as conventional fixed-size filters in this sub-block.

Secondly, the dataset to be classified is spectrograms containing m-D signatures, which might be considered weak features, which are easily filtered through Conv-unit, resulting in a decrement of classification accuracy. To address this issue, cross-residual connections are employed appropriately in im-res blocks to reuse previously useful feature maps, which may be lost when going through the model's extraction core flow.”

**7. How do the adaptable cross-residual connections in the CRCNN model contribute to its performance in classifying activities based on micro-Doppler signatures? What are the advantages of using different filter sizes in the CRCNN model, and how do they affect feature extraction?**

**Author response:** The authors would like to thank the Reviewer for your constructive comment.

***Firstly, the author explains why cross-residual connections are used, as well as their performance in classifying activities based on micro-Doppler signatures.***

Cross-residual connections have all the main advantages of residual connection (also known as skip connections), such as mitigating the vanishing gradient problem, easier optimization, and improved feature reuse. However, the cross-residual connection makes a longer connection than the residual connection due to cross-connections throughout a 1x1 conv layer. These connections are employed for continuously reusing the useful former feature maps, which can be lost when going through the model’s extraction backbone flow.

Specifically:

The output of norm 1:

The output of the im-res block 1:

where, and are convolutional operation of Conv-unit and conv layer, respectively, and $N$ is normalization operation.

The output of norm 2:

The output of the im-res block 2:

The output of norm 3:

The output of the im-res block 3:

…

The output of norm 7:

The output of the im-res block 7:

From the $2^{nd}$ im-res block. Along $N_{2}$, $\Sigma_{2}$ will accumulate the  feature from the previous norm layer. The accumulation will repeat continuously until the 7^th^ im-res block. This will add the weaker features, which may have been removed through the layers' previous conv-unit is added back. As a result, the obtained features will be richer and more diverse. (This content is presented on pages 11 and 12, section "Proposed CRCNN-based classification approach", in manuscript

**Author action 1:** The author adds the reasons to use C-R connections in Section “Proposed CRCNN-based classification approach”. The revised content is highlighted in blue on page 11 of the “Revised Manuscript with Track Changes”, as follows:

“Secondly, the dataset to be classified is spectrograms containing m-D signatures, which might be considered weak features, which are easily filtered through Conv-unit, resulting in a decrement of classification accuracy. To address this issue, cross-residual connections are employed appropriately in im-res blocks to reuse previously useful feature maps, which may be lost when going through the model's extraction core flow. Cross-residual connections have all the main advantages of residual connection (also known as skip connections), such as mitigating the vanishing gradient problem, easier optimization, and improved feature reuse. However, the cross-residual connection makes a longer connection than the residual connection due to cross-connections throughout a 1x1 conv layer.”

***Secondly, the authors present three main advantages of using different filter sizes in the CRCNN model.***

In CRCNN model, filter sizes play a crucial role in feature extraction. There are three main advantages of using different filter sizes and how they affect feature extraction:

Firstly, it enables multi-scale feature extraction by capturing details at various levels, from fine local patterns to broader global context. Different filter sizes enable the network to capture features at multiple scales. The 1x1 and 3x3 size filters are effective at extracting fine-grained features, while larger filters (5x5) capture broader features. This multiscale approach allows the network to learn features at various levels of abstraction, leading to richer representations of the input data.

Secondly, incorporating filters of different sizes enhances the robustness of the network to variations in the input data. Since different features may be best extracted by filters of different sizes, the network becomes more resilient to changes in the input, such as variations in object distance, aspect angles, or noise levels.

Thirdly, using a combination of filter sizes allows CRCNN to learn more efficiently by distributing the feature extraction task across multiple filters. Instead of relying solely on a single filter size to capture all relevant features, the network can leverage the complementary strengths of filters with different receptive fields to achieve better overall feature extraction performance.

**Author action 2:** The author summarized discusses and added in Section “Proposed CRCNN-based classification approach”. The revised content is highlighted in blue on page 11 of the “Revised Manuscript with Track Changes”, as follows:

“Firstly, the convolutional layers of conventional CNNs or DCNNs usually have a size of 3x3. However, the region containing valuable m-D signatures of various human activities on the input spectrogram has completely different distributions based on the type of activity, distance, and aspect angle of the radar. Therefore, using a filter with a fixed size of 3x3 will not be effective in extracting features, and determining the ideal filter size for extracting and learning those signatures is a challenge. A larger filter yields a global distribution, whereas a smaller one yields a distribution that is limited to a specific locality. Besides, raising the depth is susceptible to overfitting. The answer to this difficulty is to run different-sized kernels on the same input tensor [16]. Therefore, in the structure of the proposed model, filters of different sizes, including 1x1, 3×3 and 5×5, are intelligently designed to be able to extract the most diverse features, including global and local distribution from the same input feature map. Besides, the design can ensure the best balance between classification accuracy and computational complexity when using more than two conv-unit as well as conventional fixed-size filters in this sub-block.”

**Author action 3:** To discuss the impact of using filters of different sizes on feature extraction. The author added experimental results when changing the filter size in the im-res block in Section "The effect of the filter size in the im-res block on the performance of the proposed model”. The revised content is highlighted in blue on page 13 of the “Revised Manuscript with Track Changes”, as follows:

“Table 2. Compare results with different size filters in the im-res block

| Filter size | Accuracy (%) | | | | | | | Para  (M) | Train  (m) | Pre  (ms) |
| --- | --- | --- | --- | --- | --- | --- | --- | --- | --- | --- |
|  | -15dB | -10dB | -5dB | 0dB | 5dB | 10dB | avg |  |  |  |
| 3x3//3x3 | 69.39 | 83.18 | 84.55 | 87.27 | 93.64 | 95.61 | **85.61** | 0.8 | 59 | 12.9 |
| 3x3//5x5 | 74.85 | 86.21 | 87.27 | 91.61 | 96.36 | 98.33 | **89.11** | 1.2 | 61 | 13.1 |
| 3x3//7x7 | 75.76 | 85.3 | 85.61 | 90.61 | 95.3 | 97.58 | **88.36** | 1.9 | 67 | 13.7 |
| 5x5//7x7 | 73.03 | 85.91 | 86.06 | 89.55 | 95.3 | 97.88 | **87.96** | 2.4 | 71 | 14.4 |

**Note:** Para is the learnable parameters, Train is training time and Pre is the prediction time; (M denotes million; m and ms denote minute and millisecond, respectively).

Table 2 displays the comparison results for accuracy when changing the size of filters in im-res blocks. The model, which uses 2 parallel convolutional layers with a fixed size of 3x3//3x3, has the fastest prediction time, but the classification accuracy is the lowest (just 85.61%). Models using filters of sizes 3x3//7x7 and 5x5//7x7 achieve accuracies of 88.36% and 87.96%, respectively, but they come at the cost of a sharp increase in learned parameters and prediction time. With a selected size of 3x3//5x5, the CRCNN model achieved the highest accuracy (at 89.11%) and a prediction time of 13.1 ms. This result has proven that using filters with different sizes will help extract both local and global features more optimally, and 3x3 and 5x5 are the appropriate sizes for the features to be extracted from the input dataset in the proposed CRCNN model.”

**8. How does the proposed denoising technique specifically enhance the classification accuracy, and what are the key factors influencing its effectiveness?**

**Author response:** The authors would like to thank the Reviewer for your constructive comment.

In this study, we focus on two main proposals to increase classification accuracy.

First proposal: remove white Gaussian noise in the preprocessing step (from IQ raw data containing white Gaussian noise until processed into spectrograms for input to the classifier).

Second proposal: improve the CNN model to increase feature extraction capability and classification accuracy while maintaining reasonable computing costs.

In the first proposal, we focus on determining the optimal range-bin range on the range-profile matrix using the minimum entropy information criterion. Next, determine an adaptive cut-off threshold value when performing STFT on the selected optimal range-bin interval. The key factor influencing this proposal's effectiveness is how to choose an appropriate adaptive cutoff value. The cut-off threshold value was chosen based on empirical knowledge and testing on the dataset. Using a low cut-threshold value will preserve important m-D signatures, but it will also introduce substantial background noise, leading to a decrease in classification accuracy. Conversely, if the cut-threshold value is high, both noise and m-D signals will be erased. Hence, the selection of the cutting threshold value mostly impacts the quality of the proposed denoising and processing block.

The key factors influencing the second proposal's effectiveness are the selection of the number of layers, the number of filters, and the size of the filters. This selection was based on empirical knowledge and testing of the dataset. The authors have experimented with and explained in detail these selections in Section "CRCNN performance evaluation."

**Author action:** The author adds additional content in Paragraph 1 in Section “Proposed method”. The revised content is highlighted in blue on page 6 of the “Revised Manuscript with Track Changes”, as follows:

Figure 5. The diagram of the proposed approach

“The diagram of the proposed approach is given in Figure 5. The Denoising and Processing block is responsible for removing noise from the noisy raw data. The output of this block is the denoised spectrograms before being fed into the classifier. Then, a lightweight DCNN model with a design based on cross-residual connections, namely the Cross-Residual Convolutional Neural Network (CRCNN), is used to classify human activities based on m-D signatures obtained from the input denoised spectrograms.”

**9. Can you provide a more detailed analysis of the experimental results, including the impact of different noise levels on the classification accuracy?**

**Author response:** The authors would like to thank the Reviewer for your constructive comment.

We replaced image 14 (in the manuscript) by table 6 to evaluate more detail impact of different noise levels on the classification accuracy.

**Author action:** The author adds additional content in Section “Comparison results of CRCNN with other classifiers”. The revised content is highlighted in blue on pages 18 and 19 of the “Revised Manuscript with Track Changes”, as follows:

Table 6. Performance metrics of different classifiers at different SNRs on two datasets.

| Classifiers | Dataset | Accuracy (%) | | | | | | |
| --- | --- | --- | --- | --- | --- | --- | --- | --- |
|  |  | -15dB | -10db | -5dB | 0dB | 5dB | 10dB | avg |
| DIAT | Noise | 46.21 | 65.61 | 65.91 | 69.39 | 85.91 | 86.06 | 69.85 |
|  | Denoised | 93.64 | 97.27 | 98.33 | 98.64 | 99.09 | 99.24 | 97.7 |
|  | **Improved** | **47.43** | **31.66** | **32.42** | **29.25** | **13.18** | **13.18** | **27.85** |
| DINN | Noise | 46.36 | 67.58 | 68.03 | 70.61 | 88.79 | 93.64 | 72.5 |
|  | Denoised | 88.18 | 92.58 | 94.24 | 95.3 | 97.73 | 98.48 | 94.42 |
|  | **Improved** | **49.4** | **45.75** | **44.85** | **39.7** | **30.91** | **30.76** | **21.92** |
| RepVGG | Noise | 63.18 | 73.33 | 74.55 | 75.61 | 80.91 | 84.7 | 75.38 |
|  | Denoised | 94.7 | 96.82 | 97.12 | 97.27 | 97.73 | 98.79 | 97.07 |
|  | **Improved** | **31.52** | **23.49** | **22.57** | **21.66** | **16.82** | **14.09** | **21.69** |
| Dop-DenseNet | Noise | 65.91 | 80.45 | 80.76 | 83.64 | 88.33 | 93.33 | 82.07 |
|  | Denoised | 97.12 | 98.03 | 98.79 | 98.84 | 99.09 | 99.24 | 98.52 |
|  | **Improved** | **31.21** | **17.58** | **18.03** | **15.2** | **10.76** | **5.91** | **16.45** |
| MobileNet-V2 | Noise | 73.48 | 85.42 | 86.36 | 90.52 | 94.09 | 97.42 | 87.88 |
|  | Denoised | 97.42 | 98.94 | 99.24 | 99.39 | 99.48 | 99.55 | 99 |
|  | **Improved** | **23.94** | **13.52** | **12.88** | **8.87** | **5.39** | **2.13** | **11.12** |
| ResNet | Noise | 72.61 | 85.12 | 85.67 | 88.33 | 93.33 | 97.12 | 87.03 |
|  | Denoised | 97.27 | 98.79 | 99.09 | 99.24 | 99.35 | 99.39 | 98.86 |
|  | **Improved** | **24.66** | **13.67** | **13.42** | **10.91** | **6.02** | **2.27** | **11.83** |
| CRCNN | Noise | 74.85 | 86.21 | 87.27 | 91.61 | 96.36 | 98.33 | 89.11 |
|  | Denoised | 98.33 | 99.09 | 99.39 | 99.55 | 99.7 | 99.85 | 99.32 |
|  | **Improved** | **23.48** | **12.88** | **12.12** | **7.94** | **3.34** | **1.52** | **10.21** |

Table 6 shows the average classification accuracy at different noise levels for two datasets performed on six existing DCNNs and a proposed CRCNN network. According to the experimental results, classification accuracy increases as the SNR level rises. This proves that the signal-to-noise ratio directly impacts the classification accuracy of the models. In particular, Figure 14a shows that the noise-added dataset of models has a classification accuracy range of about 45% to 75% at an SNR of -15 dB. This range slowly rises to about 85% to 98% at 10 dB-SNR. For the denoised dataset (Figure 4b), the accuracy fluctuates from 88% to 98% at -15 dB-SNR and increases from 98% to 99% at 10 dB-SNR. The results show that the denoising method, which uses the appropriate cut-threshold value and the optimal range-bin interval, can effectively eliminate white Gaussian noise. Observing the models in detail, we can see that the improvement gradually decreases as the SNR level increases, which proves that the proposed method is really effective at low SNR levels and still maintains significant improvement at high SNR levels. Furthermore, for both datasets, the proposed CRCNN classification model achieves higher accuracy than the remaining models. Especially when combining the CRCNN and the proposed denoising method, the accuracy reaches over 99% at -10 dB-SNR.

**10. How do the findings of this study compare with other state-of-the-art methods in radar-based human activity classification?**

**Author response:** The authors would like to thank the Reviewer for your constructive comment.

**Author action:** The author adds additional content in Section “Comparison results on the noise-added dataset”. The revised content is highlighted in blue on pages 14 and15 of the “Revised Manuscript with Track Changes”, as follows:

“Dense-Inception Neural Network (DINN) and DIAT-RadHARNet (DIAT) are two state-of-the-art methods in radar-based human activity classification, which are published in 2022 and 2023, respectively. The authors added comparison results with the two mentioned models to further evaluate the performance of the proposed method. Additional comparison results of the two models are presented in Table 7.”

Table 7. The classification accuracy of DINN and DIAT.

| Model | Data | Accuracy (%) | | | | | | | Train  (m) | Pre  (ms) |
| --- | --- | --- | --- | --- | --- | --- | --- | --- | --- | --- |
|  |  | -15dB | -10dB | -5dB | 0dB | 5dB | 10dB | avg |  |  |
| DIAT | Noise | 46.21 | 65.61 | 65.91 | 69.39 | 85.91 | 86.06 | **69.85** | 146 | 14.6 |
|  | Denoised | 93.64 | 97.27 | 98.33 | 98.64 | 99.09 | 99.24 | **97.7** | 121 | 12.8 |
| DINN | Noise | 46.36 | 67.58 | 68.03 | 70.61 | 88.79 | 93.64 | **72.5** | 93 | 13.3 |
|  | Denoised | 88.18 | 92.58 | 94.24 | 95.3 | 97.73 | 98.48 | **94.42** | 78 | 10.7 |
| Proposed | Noise | 74.85 | 86.21 | 87.27 | 91.61 | 96.36 | 98.33 | **89.11** | 61 | 13.1 |
|  | Denoised | 98.33 | 99.09 | 99.39 | 99.55 | 99.7 | 99.85 | **99.32** | 51 | 10.6 |

**11. What are the potential limitations of the proposed approach, and how might they be addressed in future research**

**Author response:** The authors would like to thank the Reviewer for your constructive comment.

The proposed approach's potential limitations are that it does not address the classification of human activities by multiple subjects, and only additional white Gaussian noise has been considered.

To address the above limitations, future research will focus on collecting data from multiple subjects performing the same activities. This will diversify the dataset and enable the model to learn more robust features that generalize across different individuals. Further research about filters, which are used to separate m-D signatures of each individual person. Consider further the impact of different types of noise on radar signals, thereby researching filters to eliminate different types of noise, helping to improve classification accuracy.

**Author action:** The author adds additional content in Section “Conclusion”. The revised content is highlighted in blue on page 20 of the “Revised Manuscript with Track Changes”, as follows:

“Based on the aforementioned findings, future research in this area should focus on addressing the classification of human activities by multiple subjects and considering the impact of different types of noise.”

**12. What are the main implications of the study's findings for the field of human activity classification using radar sensors?**

**Author response:** The authors would like to thank the Reviewer for your constructive comment.

In the field of human activity classification using radar sensors, the two most important things that need to be achieved are high classification accuracy and low processing time. The research results have proposed a denoising method and a lightweight network model, which help solve both problems simultaneously for classifying human movements using radar.

**Author action:** The author adds additional content in Section “Conclusion”. The revised content is highlighted in blue on page 20 of the “Revised Manuscript with Track Changes”, as follows:

“In this study, a denoising filter has the task of removing white Gaussian noise from raw radar signals before classifying human activities using CRCNN are proposed to improve the classification accuracy of human activities based on m-D signatures influenced by noise.”

**13. What future directions for research are suggested by the results of this study?**

**Author response:** The authors would like to thank the Reviewer for your constructive comment.

The initial goal of the research is to classify activities from people's daily activities. Based on the findings, a number of research directions can be applied, such as:

Applied in health care: detecting and accurately classifying patients' activities can help monitor health status and promptly detect abnormal activities that are potentially dangerous, such as falls due to stroke and myocardial infarction, especially for the elderly and people with special needs.

Applied in security and military: Accurate classification of human activities via radar sensors can help in detecting and preventing security and military-threatening activities, such as unauthorized intrusion into prohibited areas, illegal border crossing, or transporting weapons.

**Author action:** The author adds additional content in Section “Conclusion”. The revised content is highlighted in blue on page 20 of the “Revised Manuscript with Track Changes”, as follows:

“The proposed method will be further optimized, verified by experimental measurement, and implemented in a real system for human activity classification applications.”
